# Chronic Viral Hepatitis in Malaysia: "Where are we now?"

**DOI:** 10.5005/jp-journals-10018-1214

**Published:** 2017-05-05

**Authors:** Ruksana Raihan, Rosmawati Mohamed, Muhammad Radzi Abu Hassan, Rosaida Md Said

**Affiliations:** 1 Department of Microbiology, Faculty of Medicine, AIMST University, Kedah, Malaysia; 2 Department of Medicine, Faculty of Medicine, University of Malaya, Kuala Lumpur, Malaysia; 3 Department of Gastroenterology Services, Ministry of Health, Kuala Lumpur, Malaysia; 4 Department of Medicine, Hospital Ampang, Ministry of Health, Kuala Lumpur, Malaysia

**Keywords:** Global target, Hepatitis B virus, Hepatitis C virus.

## Abstract

Malaysia is a country where an estimated 1 million people are chronically infected with hepatitis B virus (HBV) and an estimated 2.5% of the adult population are positive for antibody to hepatitis C virus (HCV). Effective nationwide vaccine coverage seems to be a highly effective measure to prevent new HBV infection. Treatment of HCV infection is also a regular practice in Malaysia. These measures highlight the possibility to reach the World Health Organization elimination target by 2030. To achieve this target, the Health Ministry and other nongovernmental organizations, such as My Commitment to Cure (MyC2C) are working together to develop a strategic road map to reach the global elimination target in Malaysia by 2030.

**How to cite this article:** Raihan R, Mohamed R, Hasan MRA, Rosaida MS. Chronic Viral Hepatitis in Malaysia: "Where are we now?" Euroasian J Hepato-Gastroenterol 2017;7(1):65-67.

## INTRODUCTION

Globally, around 400 million people live with hepatitis B virus (HBV) or hepatitis C virus (HCV) infection, with no country/region being left unaffected; 1.4 million people die every year from complications of viral hepatitis. There is a lack of global awareness, and most persons with hepatitis remain undiagnosed, yet most of these deaths can be prevented as there are highly effective measures to prevent new HBV and HCV infections and highly effective treatments that can suppress HBV replication and cure HCV infection. The adoption of the first ever World Health Organization (WHO) global health sector strategy on viral hepatitis (GHSS-VH) at the World Health Assembly 2016 the greatest global commitment in viral hepatitis to date, has paved the way for the elimination of viral hepatitis as a problem of public health concern, and national policies for viral hepatitis are required to reach the ambitious 2030 targets.^1^

## CURRENT SITUATION IN MALAYSIA

**Graph 1:**
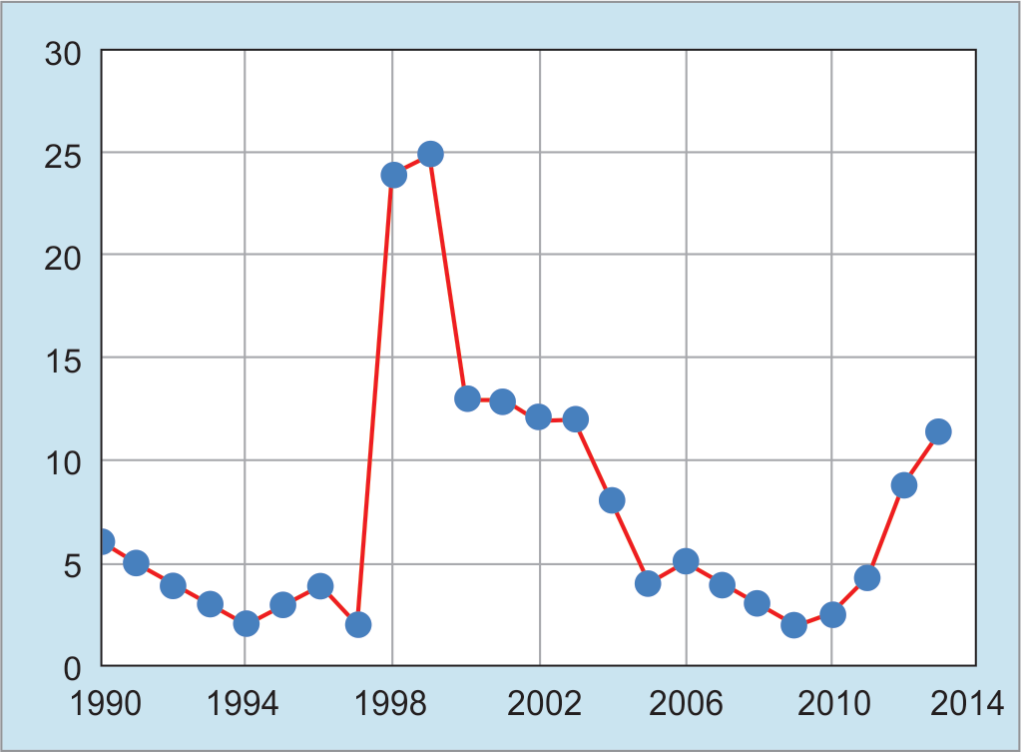
Hepatitis B virus prevalence

In Malaysia, an estimated 1 million people are chronically infected with HBV. Before the nationwide universal hepatitis B vaccination of all newborn infants since 1989, Malaysia was an intermediate endemicity country, with surface antigen of HBV (HBsAg) prevalence of 5 to 7%.^2^ In Malaysia, universal hepatitis B vaccination of all newborn infants has been implemented since 1989. The nationwide coverage for universal vaccination of HBV is about 96.29% (2014). The impact of this vaccination program on chronic HBV infection in the Malaysian population was assessed by Ng et al,^3^ where 190,077 school children aged 7 to 12 years were tested. The HBsAg positivity declined from 2.5% among those born in 1985 (before the implementation of universal infant vaccination) to 0.4% among those born in 1996. Another study by Ng et al^4^ investigated the prevalence of HBsAg among 2,923 new student intakes in the Faculty of Medicine and Dentistry in University of Malaya. The HBsAg prevalence was 1.08% (15/1390) among those born before 1989 and only 0.20% (3/1533) among those born in or after 1989. Both these findings are in support of effective prevention of HBV transmission by vaccination. In another survey of almost 900 patients with chronic HBV carried out by the Malaysian Liver Foundation, 50% of the hepatitis B carriers were HBV deoxyribonucleic acid positive, and of these, 30 to 40% have evidence of liver disease and require treatment. There are two forms of treatment currently listed in the Ministry of Health drug formulary for hepatitis B. Tenofovir, entecavir, telbivudine, lamivudine, and adefovir are forms of oral drugs available for hepatitis B treatment.^5^ Even though there’s nationwide good vaccine coverage and available treatment facilities, the increasing trend of HBV notification (Graph 1) from 2010 till now is mostly the reflection of those born before 1989, and the disease burden will remain high for some time as the infected people are getting older and living longer.

**Graph 2:**
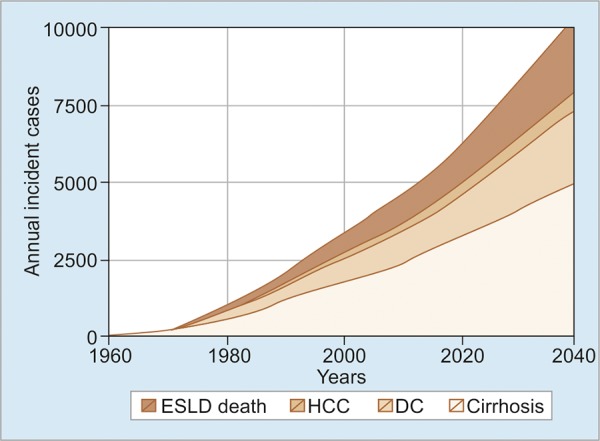
Forecasted annual incidence of cirrhosis, decompensated cirrhosis (DC), hepatocellular carcinoma (HCC) and deaths due to end-stage liver disease (ESLD) over time

Hepatitis C virus infection is a growing problem in Malaysia as more and more people are found to have a positive HCV through routine screening. The burden of HCV infection and its epidemiology in Malaysia are lacking. To estimate the prevalence of HCV, multiparam-eter evidence synthesis methods were applied where all available relevant data sources were combined, which informed the epidemiological parameters of interest. An estimated 2.5% of the adult population are anti-HCV positive and 59% (95% confidence interval: 50-68%) of which is transmitted through injection.^6^ To estimate the current and future disease burden of HCV, an age-structured multistate Markov model was developed, and the natural history of HCV infection was stimulated. From this study, it is shown that currently HCV disease burden in Malaysia is high and about to rise steeply over the coming decades (Graph 2). A cumulative total of 63,900 HCV-related deaths is projected by 2039.^7^

The development of direct-acting antiviral agents (DAAs), which are potent inhibitors of HCV replication, is a major advancement in the evolution of HCV therapy. Very high cure rates approaching 100% can be achieved with a short duration of DAA treatment. However, access to DAA remains the greatest challenge because of the high cost, and steps must be taken to address barriers to treatment.^8^

Clinical trials using generic DAA are currently ongoing due to the effort of the Ministry of Health Malaysia together with nonprofit organizations. The combination therapy ravidasvir and sofosbuvir being used in the clinical trial is estimated to cost a fraction of the currently available originator DAA regimen.^9^

## MALAYSIA IN ALIGNMENT WITH WHO TARGETS

On May 28, 2016, 194 Member States made a historic commitment to eliminate viral hepatitis by 2030. At the 69th World Health Assembly, governments unanimously voted to adopt the first ever Global Viral Hepatitis Strategy, signaling the greatest global commitment in viral hepatitis to date. The strategy sets a goal to eliminate HBV and HCV by 2030 and includes a set of prevention, diagnosis, and treatment targets, such as treatment of 80% eligible patients (Table 1), which, if reached, will reduce annual deaths by 65%, saving 7.1 million lives globally by 2030.^10^

**Graph 3:**
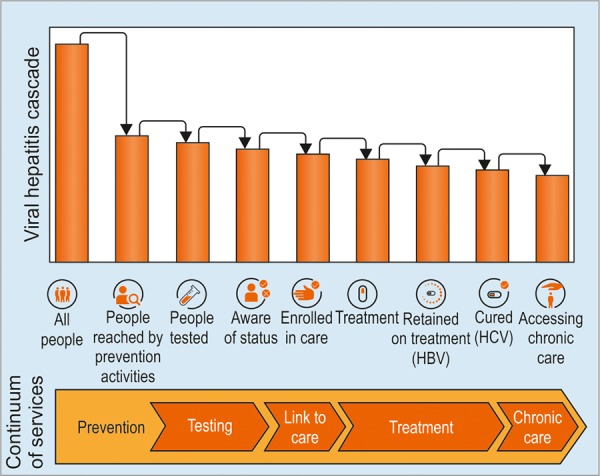
The continuum of viral hepatitis and the retention cascade. *Source:* Global Health Sector Strategy on Viral Hepatitis 2016 to 2021, Geneva, World Health Organization; 2016

The Ministry of Health in Malaysia has identified viral hepatitis as one of the priority diseases. To achieve GHSS-VH by 2030, the Health Ministry and other nongovernmental organizations, such as My Commitment to Cure (MyC2C) are working together to develop a strategic road map to reach the target in Malaysia by 2030. A national strategic plan is needed, and must include the elements mentioned in the GHSS-VH (Graph 3).

## CONCLUSION

Availability of preventive vaccine, cheaper drugs, and a comprehensive national strategic plan will help to reach the target for HBV. Besides being a curable disease and also with treatment availability, HCV remained a major concern in Malaysia as large number of people living with HCV do not know that they are infected and are often diagnosed at a late stage with manifestations of advanced cirrhosis or liver cancer. Very high cost for treatment also helped this to remain as a major concern. The high prevalence of chronic viral hepatitis with rising disease burden will require a comprehensive viral hepatitis action plan in partnership with relevant stakeholders to facilitate the development of a strategic road map to reach the GHSS-VH elimination target in Malaysia by 2030.

**Table 1:** **Table 1:** World health organization targets to eliminate HBV and HCV by 2030

*World health organization Targets*													
*Target areas*								*Baseline 2015*		*2020 target*		*2030 target*	
Service coverage		Prevention		1 Three-dose hepatitis B vaccine for infants (coverage %)				82%		92%		90%	
				2 Prevention of mother-to-child transmission of HBV: Hepatitis B birth-dose vaccination or other approaches (coverage %)				38%		50%		90%	
				3 Blood and injection safety (coverage %)		3a Blood safety: Donations screened with quality assurance		89%		95%		100%	
						3b Injection safety: Use of engineered devices		5%		50%		90%	
				4 Harm reduction (sterile syringe/needle set distributed per person per year for People who inject drugs PWIDI)				20		200		300	
				5 Treatment		5a Diagnosis of HBV and HCV (coverage %)		<5%		30%		90%	
						5b Treatment of HBV and HCV (coverage %)		<1%		5 million (HBV)		80% eligible treated	
								3 million (HCV)			
Impact leading to		Incidence of chronic HBV and HCV infections						6-10 million		30% reduction		90% reduction	
elimination		Mortality from chronic HBV and HCV infections						1.46 million		10% reduction		65% reduction
